# Bioinformatics Pipeline for Human Papillomavirus Short Read Genomic Sequences Classification Using Support Vector Machine

**DOI:** 10.3390/v12070710

**Published:** 2020-06-30

**Authors:** Alexandre Lomsadze, Tengguo Li, Mangalathu S. Rajeevan, Elizabeth R. Unger, Mark Borodovsky

**Affiliations:** 1 Wallace H. Coulter Department of Biomedical Engineering, Georgia Tech, Atlanta, GA 30332, USA; alexandre.lomsadze@bme.gatech.edu; 2 Division of High-Consequence Pathogens & Pathology, Centers for Disease Control and Prevention, Atlanta, GA 30329, USA; uyy7@cdc.gov (T.L.); mor4@cdc.gov (M.S.R.); eru0@cdc.gov (E.R.U.); 3 School of Computational Science and Engineering Georgia Tech, Atlanta, GA 30332, USA

**Keywords:** HPV typing, HPV whole genome sequencing, target enrichment, h classification, bioinformatics pipeline

## Abstract

We recently developed a test based on the Agilent SureSelect target enrichment system capturing genomic fragments from 191 human papillomaviruses (HPV) types for Illumina sequencing. This enriched whole genome sequencing (eWGS) assay provides an approach to identify all HPV types in a sample. Here we present a machine learning algorithm that calls HPV types based on the eWGS output. The algorithm based on the support vector machine (SVM) technique was trained on eWGS data from 122 control samples with known HPV types. The new algorithm demonstrated good performance in HPV type detection for designed samples with 25 or greater HPV plasmid copies per sample. We compared the results of HPV typing made by the new algorithm for 261 residual epidemiologic samples with the results of the typing delivered by the standard HPV Linear Array (LA). The agreement between methods (97.4%) was substantial (kappa = 0.783). However, the new algorithm identified additionally 428 instances of HPV types not detectable by the LA assay by design. Overall, we have demonstrated that the bioinformatics pipeline is an accurate tool for calling HPV types by analyzing data generated by eWGS processing of DNA fragments extracted from control and epidemiological samples.

## 1. Introduction

Detection and typing of the family of human papillomaviruses (HPV) in samples collected in epidemiologic studies remains an important tool for monitoring the impact of HPV vaccines. These studies provide data assuring that, of the more than 200 types currently recognized, the prevalence of types targeted by the vaccines decreases among those vaccinated appropriately, and that other types do not show an increase (type replacement). Currently, only the most common genital types (less than 40) are identified by commercially available typing assays. The advent of next generation sequencing (NGS) methods raises the possibility that questions relating to type variants, integration status, and the role of additional HPV types could be combined with vaccine monitoring studies. Recently we have developed an enriched whole genome sequencing assay (eWGS), an NGS assay for HPV using Agilent SureSelect and RNA baits covering the entire genomes of 191 HPV types to enrich the fraction of HPV target sequences [1,2]. Illumina sequencing of the enriched sample yields short genomic reads that are mapped to reference HPV genomes for type identification. This data was originally used for semi-automated HPV typing using thresholds on number of mapped reads, depth of coverage, and fraction of reference genome covered. The initially developed method was not optimized to eliminate false positive detection while maintaining sensitivity. That tedious manual curation took at least 2–3 days for up to 64 samples/run, did not leave an audit trail and could be susceptible to human error. The current project is centered on developing and testing a bioinformatics pipeline relying on open source tools to use data from the eWGS protocol [1] to identify single or multiple types of HPV present in a given sample. The main goal of the pipeline is to identify true HPV reads rapidly and automatically among the whole set that may also include reads originating from human and other contaminant species as well as HPV reads carrying erroneous indices. One of the most challenging parts of the problem is to accurately detect and classify HPV types present in low concentrations. A core classification element of the pipeline is a support vector machine (SVM) based algorithm with structure and parameters determined through analysis of short reads generated by sequencing of artificially constructed samples with known HPV types and concentrations.

## 2. Materials and Methods

### 2.1. Reference Genome Sequences

Sequences of 286 HPV genomes, 183 types recognized by the International Committee on Taxonomy of Viruses and 103 candidate types, were obtained from the Papillomavirus Episteme Database (PAVE) in February 2018 [3]. All other sequences were retrieved from NCBI [4], including a reference assembly of human genome (assembly GRCh38.p12), a sequence of beta globin gene amplified as control in the eWGS assay (accession GU324922), phage *phiX*174 (accession NC_001422), used as a control in Illumina sequencing runs, and *Escherichia coli*, an anticipated contaminant in biologic samples.

### 2.2. Epidemiological and Control Samples

We used short read sequences from eWGS of 383 samples divided into four sets (Table 1) containing 122 control/designed samples and 261 epidemiologic samples with prior typing results from Linear Array HPV Genotyping Test (LA; Roche Diagnostics, Indianapolis, IN, USA) performed as previously described [1]. We used four data sets to account for run-to-run variations in eWGS data. Data from Sets 1 and 2 were recently used in publication of the eWGS method [1] and in establishing a reproducibility of eWGS method and its limit of detection [2]. The control/designed positive samples included cell line DNAs (HeLa ~50 copies of HPV18/cell, SiHa ~1–2 copies of HPV16/cell, and CaSki ~500 copies of HPV16/cell), and HPV plasmids. In Set 1, plasmids of the 9 HPV vaccine types (HPV6, -11, -16, -18, -31, -33, -45, -52, -58) were included as individual types (50,000 copies/sample). In each sample of Set 2, each of the nine types was present in equal concentration (e.g., in Sample 1 we had 625 copies of a plasmid of each HPV type). The plasmids concentrations of each type varied from 625 to 1 copies/sample. Set 2 was assayed in two experiments, each with two replicates (total of 4 replicates). Plasmids for 18 HPV types (ten alpha HPV types: HPV6, -11, -16, -18, -31, -33, -45, -52, -53 and -58; seven beta HPV types: HPV5, -8, -15, -20, -23, -24, -36 and one gamma HPV type: HPV48) were included in Set 3 with each plasmid at 625 copies/sample. Human placental DNA and water served as HPV negative controls. A total of 261 residual extracts (input/reaction ranged from 10–100 ng) from epidemiologic studies were included in the combined data sets (15 self-collected cervicovaginal swabs (data set 1), 50 male external genital swabs (Set 3) and 196 cervical cells in PreservCyt (Set 4)). 

### 2.3. Sequencing

All samples were sequenced using the earlier published eWGS method [1]. The RNA bait library was created using 191 reference HPV genomes as well as segments of human beta-globin gene as a control for sample integrity [1]. All samples were sequenced by a two-lane flow cell by Illumina HiSeq 2500 sequencer at the Center for Disease Control and Prevention (CDC). Sequencing produced 100 nt long paired reads. Sets 1 and 2 were sequenced with 16 samples per flow cell lane while Sets 3 and 4 were sequenced with 32 samples per flow cell lane. Each sample was barcoded with a unique 8 nt long index. Sequenced data was demultiplexed using Illumina BCL2fastq software with no mismatch allowed in barcode at demultiplexing.

### 2.4. The Typing Algorithm: Outline and Initial Steps

**Figure 1:**
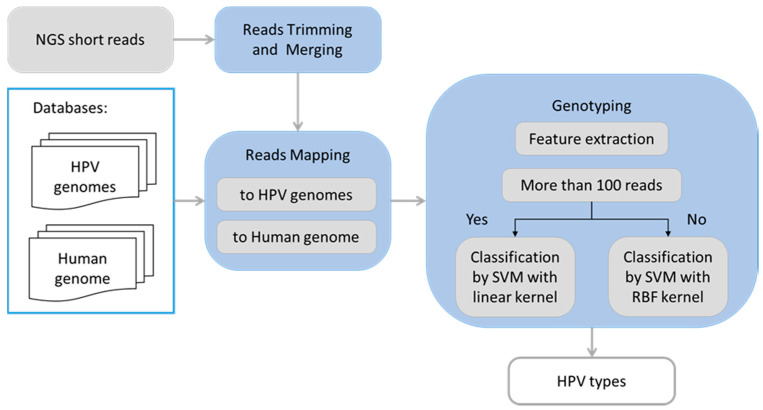
Flowchart illustrating logical steps of the human papillomavirus (HPV) typing pipeline.

The HPV typing pipeline (Figure 1) takes as input raw read pairs (from forward and reverse strands of an insert) in FASTQ format as they appear after the demultiplexing step. The read pairs were trimmed to remove low quality bases (Phred score < 4) as well as adapter sequences. If a read pair contained an overlapping section longer than 20 nt, the two reads were merged into a single read using the AdapterRemoval2 software [5].

The STAR read alignment algorithm [6] was used in a single read mode (with no intron mapping option) to align processed reads to the exclusion database (Human genome, PhiX174, and *E. coli*), allowing only 1% sequence mismatch per read. These reads were filtered out. Information about reads aligned to the globin gene (2041–3480 segment) that was used as sample control was recorded at this step. 

In the next step, the remaining reads were mapped by the STAR algorithm to reference genomes of HPV types. To account for possible HPV variants, the alignment allowed up to 10% sequence mismatch. To account for sequences that may be split as the circular HPV genome is linearized, we used duplicated sequences of the reference HPV genomes. Reads simultaneously aligned to genomes of several reference HPV types were filtered out.

The read pairs from each sample were grouped by their mapped HPV type and each group was analyzed independently using a machine learning pattern recognition algorithm implementing a well-established support vector machine (SVM) approach [7]. For supervised training of the algorithm parameters we used eWGS data from the control/designed samples. We evaluated multiple quantitative features characterizing the read pair alignments and selected four specific features.

We introduced the rate of distinct read pairs to help identify false detection due to index swapping that occurs as a side effect of parallel sequencing of several samples in the same Illumina flow lane [8]. The eWGS protocol includes a few cycles of PCR amplification so read pairs originating from a type-specific HPV DNA insert in a given sample will be replicated and are likely to be found in several copies. On the other hand, read pairs detected in the Illumina output through index swapping are likely to appear in single copies.

To clarify this point, let assume that a total number of read pairs is

*N* = *N*_1_ + 2*N*_2_ + 3*N*_3_ + … + *k* × *N_k_*

Here *N*_1_ is the number of unique read pairs, *N*_2_- the number of read pairs repeated twice, etc., and *N_k_* the number of read pairs repeated the largest number of times—*k*. Then, the number of distinct read pairs is

*M* = *N*_1_ + *N*_2_ + *N*_3_ + … + *N_k_*

Now, the rate of distinct read pairs is defined as *M/N*.

In what follows when we refer to a number of read pairs mapped to a genome of particular HPV type, we mean the number of distinct read pairs. 

The following features were used as input to the SVM classifier:

1. total number of aligned read pairs mapped to a given HPV genome
2. average depth of alignment of the read pairs to the given HPV genome
3. average coverage of the given HPV genome by aligned read pairs
4. rate of distinct read pairs

**Figure 2:**
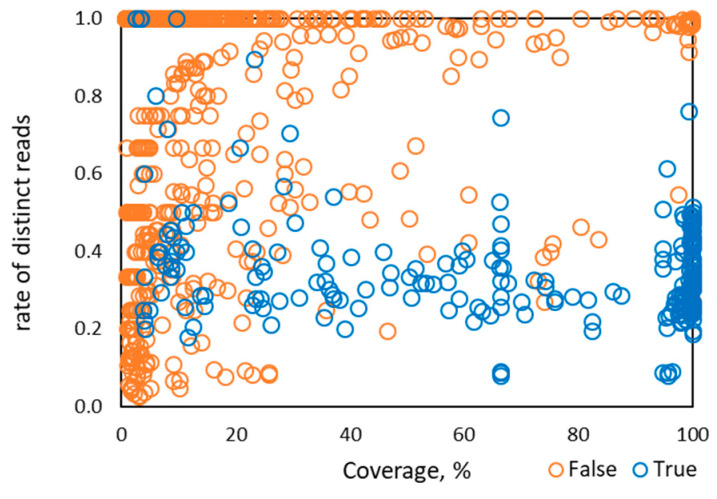
Distribution of the rate of distinct read pairs vs percentage of HPV genome coverage observed for 1286 instances of HPV types from 122 control samples in Sets 1–4.

The rate of distinct read pairs for HPV types known to be in the designed and control samples (i.e., true detection) in Sets 1–4 was largely in the range of 0.1 to 0.6 (Figure 2). Therefore, when the rate of distinct read pairs for a given HPV is close to 1.0, false detection from index swapping is likely.

### 2.5. Pattern Recognition Module of the HPV Typing Pipeline

A pattern recognition algorithm must distinguish between reads originating from an HPV type truly present (true positive reads) and those originating from assay noise (false positive reads). The read data from control/defined samples with known HPV type composition are used to determine parameters of the pattern recognition algorithm that best discriminate between true and false reads and retain high sensitivity (i.e., unlikely to miss low copy numbers of HPV truly in the sample). The four feature vectors from the whole training data set are divided into positive feature vectors from HPV types known to be present (true positive) and negative feature vectors or those that would identify an HPV type known to be absent (false positive). The process of training an SVM method is used to find a separation surface dividing the feature space into positive and negative sub-spaces.

Within the 122 samples used in training (from Sets 1–4), 28 were HPV negative while 94 included one or more HPV types resulting in a total of 263 instances of known HPV types. The mapped reads identified 256 true HPV type instances and 1030 false HPV type instances. The seven true HPV instances with no mapped reads occurred in samples with the lowest HPV concentrations. The feature vectors with 256 true and 1030 false labels were used in the classifier training and testing. 

We used an open source package LibSVM [7] (csie.ntu.edu.tw/~cjlin/libsvm, see Supplementary Materials). The SVM training and testing was done with the LibSVM default settings that required the feature vectors components to be in range (0,1). The number of reads and the depth values that varied from one to thousands were scaled into (0,1) range. The values of coverage and distinct read pair rate were within (0,1) range.

For the radial basis function (RBF) kernel, we had to select two parameters (C and γ). The selection procedure was done by five-fold splitting of the whole feature vector data into training and development sets and running an exhaustive search on a grid in the C and γ space (Figure S1). The C and γ parameters determined to be optimal were used for re-training the RBF kernel SVM on the full set of feature vectors. The SVM training was done with a goal of minimizing the false negative rate of HPV type detection while keeping the false positive rate close to zero.

**Figure 3:**
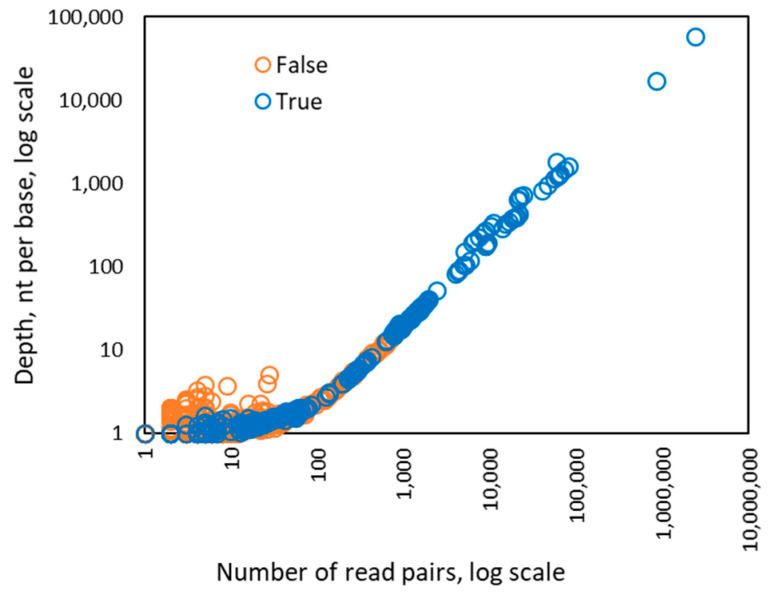
Dependence between the number of read pairs and the depth value observed for 256 instances of HPV types from 122 control samples in Sets 1–4. The dependence of the depth from the number of reads becomes linear if there are more than 100 read pairs mapped to a genome of particular HPV type.

**Figure 4:**
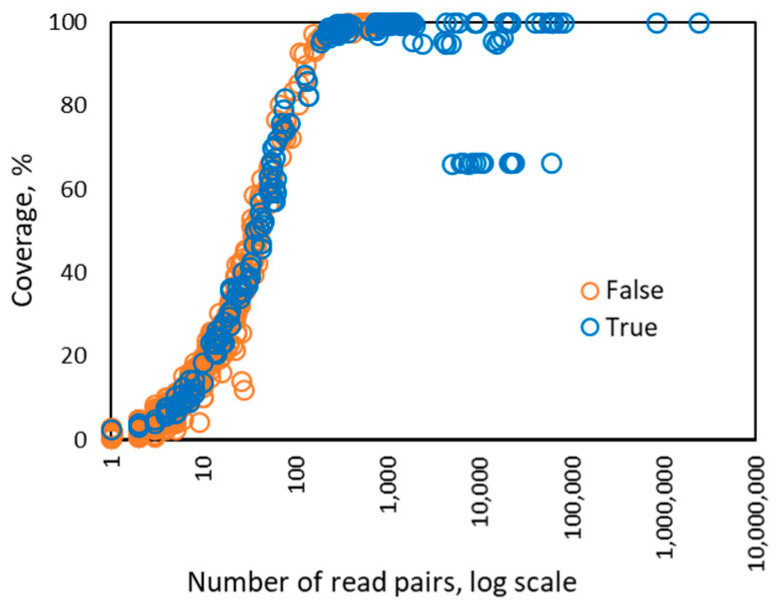
Dependence between the number of read pairs and the coverage percentage observed for 256 instances of HPV types from 122 control samples in Sets 1–4. If there are more than 100 read pairs mapped to a genome of particular HPV type, then the coverage reaches maximum value, 100%, and does not change. The points observed at near 10,000 read pairs, showing ~65% coverage may correspond to integration of HPV into human genome in HeLa originated samples.

**Figure 5:**
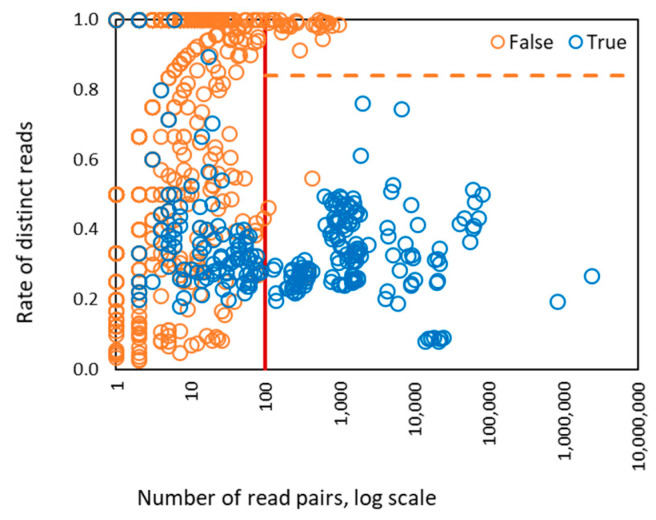
The values of two SVM features derived for 1286 HPV types present in control/designed samples. There were 186 types with >100 (de-duplicated) reads mapped to genome, 400 types with >10 read pairs mapped to genome, and 886 types with <10 read pairs mapped to genome. The vertical red line shows the separation between zones of operation of four features SVM and two features SVM. The horizontal dashed line is a separation line defined by two features linear kernel SVM for classification of true and false HPV types. The right part of the graph where the number of read pairs is >100 shows separation of HPV types classified as true and false. In the left part of the graph the separation is impossible to view in 2D plane, as it requires four-dimensional space.

Interestingly, when the number of the read pairs mapped to the genome of a particular HPV type was above 100, we observed that the value of the depth feature became linearly dependent on the number of read pairs (Figure 3). In addition, the coverage value approached 100% when the number of mapped read pairs was above 100 and stayed at that level as the number of mapped reads increased (Figure 4). Therefore, for HPV types having more than 100 mapped read pairs, those present at relatively high copy number, the optimal algorithm was a linear kernel SVM using only two features: the number of read pairs and the distinct read pairs rate. Remarkably, the separation line of the linear kernel SVM trained on mapped read data from the control/design samples showed little dependence on the number of read pairs (the dashed line in Figure 5).

For instances of HPV types with 100 or less read pairs the RBF kernel SVM used all four features. The threshold of 100 was to some extent arbitrary as in the vicinity of this threshold both methods worked with about the same level of accuracy (data not shown). For each sample, read pairs mapped to a particular HPV type were processed independently of those mapped to other HPV types and the decision to use 4-feature or 2-feature SVM was made for each type. 

### 2.6. Validation of Pipeline Classification

The optimized bioinformatics pipeline was applied to the 261 epidemiologic samples (Sets 1, 3, 4) previously assayed with LA. For the 37 types detected by LA, type-specific agreement rate and kappa coefficients (k) between LA and the eWGS pipeline results were calculated using GraphPad [9]. Agreement between methods was interpreted as poor if kappa value k < 0.20, fair, for 0.21 < k < 0.40, moderate, for 0.41 < k <0.6, substantial, for 0.61 < k < 0.80 and almost perfect if 0.81 < k <1.00. As an indication of HPV types that would be missed by LA, we also report the full list of all types identified in eWGS data (Table S3). 

## 3. Results

### 3.1. Automated HPV Typing by a Bioinformatics Pipeline: Control/Designed Samples (n = 122)

Detection and typing accuracy of the SVM based bioinformatics pipeline was evaluated in the control/designed samples used for SVM training. In Set 1, prepared with high HPV copy numbers, all HPV types were correctly detected without false positive predictions. In sample 11 from this set containing Caski DNA we observed the highest HPV copy number, resulting in 2,403,579 HPV16 read pairs. Notably, HPV16 was detected in all samples in the same flow cell lane, including negative controls (placenta and water, samples 10 and 9), but were not detected in the second lane of the flow cell. Presence of HPV16 false reads could be explained by index swapping [8], and the pipeline correctly assigned these reads as false. The distinct read pair rate was a key feature for discriminating between true reads and reads originating from index swapping. Indeed, if the SVM classifier was trained without the rate of distinct read rate feature, a high false positive rate was observed (data not shown).

Set 2 included four replicates designed to simulate infection with multiple HPV types per sample and to determine reproducibility and the limit of detection of eWGS assay [2]. As shown in Table 2, the SVM-based pipeline detected all true HPV types with no false positive results for samples with 25 or greater HPV plasmid copies per sample. However, for samples prepared with plasmids at lower concentrations (five and one HPV plasmid copy per sample), the pipeline failed to detect HPV types in any samples having only one HPV copy, and also failed to detect 16 of the 36 instances of HPV types samples having five HPV plasmid copies. In seven of the 52 instances of missed HPV types (false negatives) no reads of the expected type were mapped to a relevant HPV genome. In the remaining 45 instances, reads of the expected type were present (in the range from 1 to 20 reads), but were not classified as “true” by the algorithm. In a few instances of HPV types 16 and 18, the number of reads was above 10 but a ‘leaking’ (due to index swapping of reads from the same HPV types present in other samples in the same Illumina lane) overshadowed the presence of the true reads and the algorithm called the reads from these types ‘false’ (Tables S1 and S2). Notably, even in the low copy number samples, the pipeline gave no false positive results.

The total number of instances of known HPV types in designed samples of Sets 3 and 4 was 52 (38 in Set 3 and 14 in Set 4). The pipeline correctly detected all 52 instances of HPV types (Table 3). However, it also detected eleven additional instances of HPV types that should not be present (false positives). HPV3, HPV31 and HPV53 were the most frequent types falsely detected. Index swapping was excluded as an origin of the error as there were no samples with these HPV types in high concentration in the same lanes of the flow cell. Small numbers of reads mapped to more than 30 HPV types classified as “false” by the typing pipeline could not be attributed to index swapping. Reads from human sequences with regional similarities to some HPV types, i.e., HPV types -71, -29, -77, -118 and -92 may be another source of noise; however, the origin of some low intensity noise could not be determined.

### 3.2. Automated HPV Typing by Bioinformatics Pipeline: Epidemiological Samples (n = 261)

**Figure 6:**
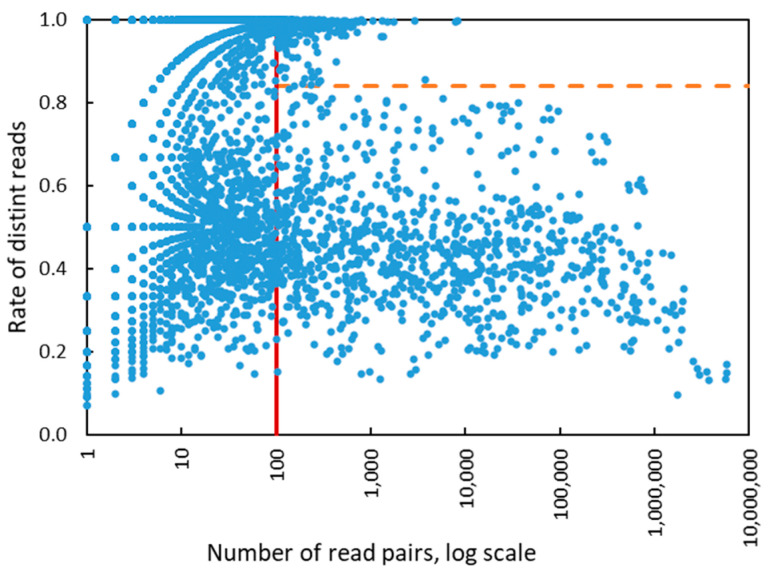
The values of two SVM features derived for 6848 HPV types present in all epidemiological samples. There were 1124 types with >100 (de-duplicated) reads mapped to genome, 2631 types with >10 read pairs mapped to genome and 4217 types with <10 read pairs mapped to genome. The vertical red line shows the separation between zones of operation of four features SVM and two features SVM. The horizontal dashed line is a separation line defined by two features linear kernel SVM.

The eWGS results for the 261 epidemiological samples (Sets 1, 3, 4) gave 6848 instances of HPV types by mapped reads. Of these, 1124 instances fell within the 2-feature SVM algorithm and 4217 required the 4-feature SVM (Figure 6). We evaluated the overall HPV type-specific concordance between the bioinformatic pipeline results and results of LA typing (Table 4). Restricted to the 37 LA types, type-specific agreement was 97.4% with *k* = 0.783 (substantial agreement). The proportion of positive agreement was 80% and proportion of negative agreement 98.5%. 

There were 117 instances in which HPV detected by LA were not identified by eWGS assay pipeline. Among these were 31 instances with no reads and 68 instances with less than 100 reads mapped to the expected HPV type. In the remaining 18 instances in which more than 100 reads were mapped, the SVM algorithm missed particular HPV types because the distinct read pairs rate was slightly above the linear kernel SVM separation line (Figure 6).

Among the 133 discordant instances in which the eWGS assay detected an HPV not identified by LA, 42 had more than 500 reads mapped to the HPV type. On the other hand, there were 22 instances in which the eWGS assay detection was based on less than 100 mapped reads. The eWGS assay detected 176 different HPV types, including an additional 428 instances of HPV types not included in the LA assay (Table S3). The 176 types include 40 that were not covered by the Agilent designed baits. 

Data from Sets 1–3 were previously analyzed manually using threshold values in number of mapped reads, depth of coverage, and percentage coverage [2]. The automated SVM classifier improved the lower limit of detection, shown most clearly in results from Set 2 where the limit for reliable detection of HPV plasmids changed from the previous limit of 125 copies to 25 copies (Table 2).

The SVM training on a set of 1000 samples was taking several minutes. This time was insignificant in comparison with total time required for analysis of a set of samples. Calculation of the SVM features for a single sample from raw reads did take ~5 min. Therefore, for example, the time needed for analysis of the whole Set 3 with 64 samples on one CPU is about 5 h.

## 4. Discussion

Our results demonstrated that eWGS data generated for HPV typing could be efficiently processed by a new machine learning method that employed an SVM classification algorithm in a fully automated pipeline. The pipeline uses open source tools that will make it easier for others to adopt. Compared with the manual method that relied on simple thresholds [2], the automated SVM classified provided better noise modeling, improved the limit of detection, and could be completed in a few hours, compared with several days. Automation also reduces error associated with manual data manipulation. Both experimental (eWGS) and computational components of the HPV typing method described here could be generalized for typing of other DNA viruses. 

The SVM parameters were developed using eWGS data from 122 defined/control samples used as training sets. The algorithm was initially examined using the defined/control sample data and further validated using eWGS data from 261 epidemiologic samples with prior typing results (Table 4). The algorithm made 100% accurate predictions (with no false positive or false negative HPV types) in the control samples with HPV copy number of 25 or greater (Table 2). Only in the case of very low HPV copy numbers, such as 5 and 1, could the method not detect HPV types present in the control samples, thus generating false negative results (Table 2). In the epidemiologic data sets, the type-specific agreement with prior data was 97.4% (Table 4).

The Illumina read pairs from the eWGS assay were processed by removing those that could originate from the human genome or *E. coli*, an anticipated contaminant. After mapping the remaining read pairs to the genomes of 286 HPV types those reads that had equal similarity to genomes of two different HPV types were eliminated as well.

The read pairs that appeared as an input to the SVM algorithm still contained two types of noise

i. HPV read pairs from one sample incorrectly assigned to another sample (index swapping);
ii. read pairs similar to some HPV genomes but originated from unknown sample contamination.

In our experiments, index swapping was observed to be the main source of noise complicating HPV typing. In a prior published study, the index swapping (bleeding) rate in multiplexed sequencing experiments was estimated to be ~0.3% [8], similar to the rate we observed (data not shown). To avoid the large number of false positive HPV predictions due to index swapping, we introduced the rate of distinct read pairs as a component of the SVM feature vector. In a more general context, this feature should be useful for methods that use Illumina sequencing to detect other infectious agents. The effectiveness of this feature depends on the experimental setup, particularly on the number of PCR cycles used in a library preparation. To avoid redundant and potentially biased sequencing the eWGS method used a relatively low number of PCR cycles, 14, to amplify inserts prior to sequencing. This assay design gave a two-fold read duplication rate that was sufficient for use of rate of distinct reads and accurate functioning of the pipeline [9]. 

HPV3 was not used in any design/control samples but was the most frequently detected among HPV types that were not supposed to be present. The same HPV3 sequence, with characteristic single nucleotide polymorphisms, was present in epidemiological samples. The presence of HPV3 as a contaminant is likely. While most false positive results in control samples could be explained by index swapping, others could be due to sample contamination by extraneous reads with similarity to parts of some HPV genomes.

Interestingly, the number of read pairs larger than ~300 per HPV genome was sufficient to cover and assemble the full genome (Figure 4). In such cases, the HPV type could further be classified as known or new variant/lineage or sub-lineage or even as a new type. Deep sequencing (with more than 8000 read pairs per an HPV genome) also provided an opportunity to detect HPV integration into the human genome (Figure 4). However, a sparse representation of an HPV type by mapped reads would lower the resolution of the variant/sub lineage/integration identification. The variant identification and detection of HPV integration will be subjects of future work. 

## Figures and Tables

**Table 1:** Description of control/designed samples (shaded cells) and epidemiological samples used to generate Data Sets 1–4.

Data Set 1 (*n* = 32)			Data Set 2 (*n* = 64)		
Sample ID	Sample Description	Input/Reaction	Experiment 1 Sample ID	Sample Description	Input/Reaction
1	HPV-plasmid-45	50,000 copies	1	Pool of HPV plasmid-11,16,31,45,52	625 copies
2	HPV-plasmid-58	50,000 copies	2	Pool of HPV plasmid-11,16,31,45,52	125 copies
3	HPV-plasmid-31	50,000 copies	3	Pool of HPV plasmid-11,16,31,45,52	25 copies
4	HPV-plasmid-33	50,000 copies	4	Pool of HPV plasmid-11,16,31,45,52	5 copies
5	HPV-plasmid-52	50,000 copies	5	Pool of HPV plasmid-11,16,31,45,52	1 copy
6	HPV-plasmid-6	50,000 copies	6	Pool of HPV plasmid-6,18,33,58	625 copies
7	HPV-plasmid-18	50,000 copies	7	Pool of HPV plasmid-6,18,33,58	125 copies
8	HPV-plasmid-11	50,000 copies	8	Pool of HPV plasmid-6,18,33,58	25 copies
9	H_2_O (HPV negative)	0 ng	9	Pool of HPV plasmid-6,18,33,58	5 copies
10	Placenta (HPV negative)	100 ng	10	Pool of HPV plasmid-6,18,33,58	1 copy
11	CaSki (HPV-16)	100 ng	11	HPV plasmid-16	10,000 copies
12	CaSki (HPV-16)	10 ng	12	HPV plasmid-18	10,000 copies
13	SiHa (HPV-16)	100 ng	13	H_2_O (HPV negative)	0 ng
14	SiHa (HPV-16)	10 ng	14	Placenta (HPV negative)	100 ng
15	HeLa (HPV-18)	100 ng	15	SiHa (HPV-16)	10 ng
16	HeLa (HPV-18)	10 ng	16	HeLa (HPV-18)	10 ng
17–31	Epidemiological samples (50 extracts from male genital swab)	100 ng	17–32	Replicate of 1–16	
32	HPV-plasmid-16	50,000 copies	Experiment 2 Sample ID	Repeated as in Experiment 1	
**Data Set 3 (*n* = 64)**			**Data Set 4 (*n* = 224)**		
**Sample ID**	**Sample Description**	**Input/Reaction**	**Sample ID**	**Sample Description**	**Input/Reaction**
15	H_2_O (HPV negative)	0 ng	15	H_2_O (HPV negative)	0 ng
16	SiHa (HPV-16)	10 ng	16	SiHa (HPV-16)	10 ng
31	Placenta (HPV negative)	100 ng	31	Placenta (HPV negative)	100 ng
32	Pool of HPV plasmid-11,16,31,45,52	625 copies	32	HeLa (HPV-18)	10 ng
44	Pool of HPV plasmid-5,8,23,36	625 copies	47	H_2_O (HPV negative)	0 ng
45	Pool of HPV plasmid-6,16,20,24,36,58	625 copies	48	SiHa (HPV-16)	10 ng
46	Pool of HPV plasmid-5,11,15,45,52	625 copies	63	Placenta (HPV negative)	100 ng
47	H_2_O (HPV negative)	0 ng	64	HeLa (HPV-18)	10 ng
48	SiHa (HPV-16)	10 ng	IDs: 1–14, 17–30, 33–46, 49–62	Epidemiological samples (56 extracts from cervical cells in PreservCyt)	Total 196 epidemiological samples in data set 4; sample input ranged from 25–100 ng
61	Pool of HPV plasmid-15,20,24,48	625 copies	IDs 65–128 *	The same order as in 1–64	
62	Pool of HPV plasmid-8,18,23,31,33,48,53	625 copies	IDs 129–192 *	The same order as in 1–64	
63	Placenta (HPV negative)	100 ng	IDs 193–224 *	The same order as in 1–32	
64	Pool of HPV plasmid-6,18,33,53,58	625 copies			
IDs: 1–14, 17–30, 33–43, 49–60	Epidemiological samples (50 extracts from male genital swab)	10–100 ng			

* Control samples included as part of these replicates.

**Table 2:** Typing accuracy for replicates of control samples from Set 2. The numbers in the table show how many times a particular HPV type was correctly identified in the four replicas of the experiment. There were no false positive predictions.

HPV Types in Samples	HPV Copy Number in Samples				
	625	125	25	5	1
HPV-11	4	4	4	2	0
HPV-16	4	4	4	3	0
HPV-31	4	4	4	2	0
HPV-45	4	4	4	4	0
HPV-52	4	4	4	2	0
False positives	−	−	−	−	−
HPV-6	4	4	4	0	0
HPV-18	4	4	4	1	0
HPV-33	4	4	4	3	0
HPV-58	4	4	4	3	0
False positives	−	−	−	−	−

**Table 3:** HPV typing accuracy in control samples in Sets 1–4.

Dataset ID	Ture HPV Instances	Correctly Detected	False Positives
Set 1	15	15	0
Set 2	196	144	0
Set 3	38	38	2
Set 4	14	14	9
Total	263	211	11

**Table 4:** Type-specific concordance between the results of HPV identification by the support vector machine (SVM) pipeline and the LA method for epidemiologic samples (the analysis is restricted to the 37 LA types).

Data Set			LA Results		Total	Agreement (%, K)	Sensitivity (%)	Specificity (%)
			+	−				
Set 1 (*n* = 15)	NGS Results	+	48	11	59	93.33 (518/555); *k* = 0.684 (95% CI 0.590–0.779) (substantial) *p* = 0.0214	65 (48/74)	97.7 (470/481)
		−	26	470	496			
	Total		74	481	555			
Set 3 (*n* = 50)	NGS Results	+	43	21	64	97.5 (7072/7252); *k* = 0.803 (95% CI 0.775–0.831) (substantial) *p* = 0.1172	78 (43/55)	98.8 (1774/1795)
		−	12	1774	1786			
	Total		55	1795	1850			
Set 4 (*n* = 196)	NGS Results	+	399	101	500	98.2 (1817/1850); *k* = 0.714 (95% CI 0.620–0.807) (substantial) *p* = 0.1627	83.4 (399/478)	98.5 (6673/6774)
		−	79	6673	6752			
	Total		478	6774	7252			
All epidemiological samples combined (*n* = 261)	NGS Results	+	490	133	623	97.4 (9407/9657); *k* = 0.783 (95% CI 0.757–0.809) (substantial) *p* = 0.3472	80.7 (490/607)	98.5 (8917/9050)
		−	117	8917	9034			
	Total		607	9050	9657			

## Supplementary Materials

The following are available online at https://www.mdpi.com/1999-4915/12/7/710/s1. Figure S1: Dependence of the SVM with RBF kernel classifier accuracy measure, proportional to (Sn + Sp)/2 on parameters C and γ. Table S1: Raw numbers of HPV reads obtained for Set 2 samples with mixture of HPV plasmids, each at 5 copy number. Table S2: Raw numbers of HPV reads obtained for Set 2 samples with mixture of HPV plasmids, each at 1 copy number. Table S3: HPV types detected in epidemiological samples.
