# Supplementary material for: Bioinformatics Pipeline for Human Papillomavirus Short Read Genomic Sequences Classification Using Support Vector Machine
Source: Viruses. 2020 Jun 30;12(7):710. doi: 10.3390/v12070710 (PMC7412107; doi:10.3390/v12070710)
Supplement: Supplementary file 1 [file viruses-12-00710-s001.pdf]

Supplementary Material for "Bioinformatics pipeline for human papillomavirus short read genomic sequences classification using support vector machine"

Alexandre Lomsadze[1], Tengguo Li[2], Mangalathu S. Rajeevan[2], Elizabeth R. Unger[2] and Mark Borodovsky[1,3*]

[1] Wallace H. Coulter Department of Biomedical Engineering, Atlanta, Georgia, USA
[2] Division of High-Consequence Pathogens & Pathology, Centers for Disease Control and Prevention, Atlanta, Georgia, USA
[3] School of Computational Science and Engineering, Georgia Tech, Atlanta, Georgia, USA
* Corresponding author: borodovsky@gatech.edu

Code availability

Developed in this project software tool, with name VirusTyping, is available at https://github.com/gatech-genemark/VirusTyping

VirusTyping is distributed under the GPL license.

SVM classifier

The read-mapping module of the pipeline attempts to align DNA reads to reference genomic sequence of each HPV type. As a result, groups of DNA reads aligned to a particular HPV genome are produced. Then we generate vectors of 'feature' values characterizing each non-empty groups for candidate HPV types. The candidates are labeled as true (predicted to be present in the sample) or false (predicted to be absent in the sample) by the SVM classifier. Several controlled samples were used for estimating classifier parameters (SVM training) as well as for evaluating classifier performance.

We used an open source package LibSVM (csie.ntu.edu.tw/~cjlin/libsvm) providing general purpose SVM implementation. SVM training and testing did use default settings.

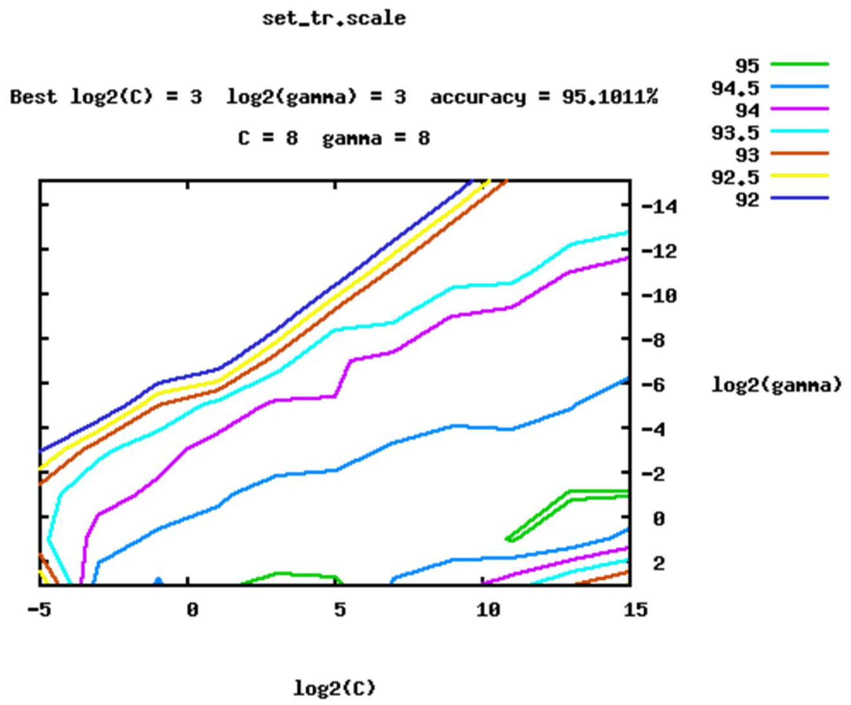

Figure S1. Dependence of the SVM with RBF kernel classifier accuracy measure, proportional to (Sn+Sp)/2 on parameters C and γ.

Particularly, values of SVM features were scaled into a fixed range, usually -1 to +1 or 0 to 1. The *coverage* and *distinct reads rate* features had values in 0 to 1 range by definition. Values of *depth* and *number of reads* features had range from 1 to thousands. The scaling was done for SVM kernels independently.

In case of RBF kernel, two parameters had to be selected (C and γ). The selection was done by 5-fold splitting of the data into training and test sets and running exhaustive search on a grid (Figure S1).

Parameters determined to be optimal were used for re-training SVM on a full set of controlled samples.

Human globin gene control

The pipeline identified the presence of the human globin gene region, used as a positive control for the eWGS assay in all samples (average 25,700 reads; range 1,500 – 96,000), except the 15 water controls prepared without human DNA that had on average three beta globin reads mapped per sample.

Table S1. Raw numbers of HPV reads obtained for Set 2 samples with mixture of HPV plasmids, each at 5 copy number.

HPV type	Replicate				Average
	1	2	3	4	
11	14	15	10	7	11.5
16	19	15	25	24	20.8
31	16	14	8	8	11.5
45	40	41	20	20	30.3
52	20	20	6	4	12.5
6	7	6	4	2	4.8
18	10	19	26	23	19.5
33	13	17	13	12	13.8
58	13	16	25	27	20.3

Table S2. Raw numbers of HPV reads obtained for Set 2 samples with mixture of HPV plasmids, each at 1 copy number.

HPV type	Replicate				Average
	1	2	3	4	
11	4	5	2	2	3.3
16	14	13	4	3	8.5
31	5	5	2	5	4.3
45	4	5	8	7	6.0
52	-	-	-	-	-
6	-	-	5	5	5.0
18	6	17	2	2	6.8
33	-	1	7	6	4.7
58	7	3	6	6	5.5

Table S3. HPV types detected in epidemiological samples.
Types in bold font were include in RNA bait library, types in shaded boxes are included in the LA assay. Dash indicates no instances in which type was detected.
* PaVE 34 corresponds to LA 64, PaVE 44 to LA 55, PaVE 82 to LA IS39, PaVE 89 to LA CP6108.

**HPV types in the reference database for read mapping** [PaVE nomenclature]	**Instances of detection by eWGS and pipeline**
**HPV_1**	-
**HPV_2**	-
**HPV_3**	30
**HPV_4**	4
**HPV_5**	3
**HPV_6**	14
**HPV_7**	-
**HPV_8**	6
**HPV_9**	1
**HPV_10**	2
**HPV_11**	6
**HPV_12**	5
**HPV_13**	-

**HPV_14**	2
**HPV_15**	-
**HPV_16**	84
**HPV_17**	-
**HPV_18**	13
**HPV_19**	1
**HPV_20**	-
**HPV_21**	1
**HPV_22**	6
**HPV_23**	5
**HPV_24**	7
**HPV_25**	1
**HPV_26**	3
**HPV_27**	1
**HPV_28**	2
**HPV_29**	-
**HPV_30**	10
**HPV_31**	40
**HPV_32**	3
**HPV_33**	15
**HPV_34 [formerly HPV 64]**	12
**HPV_35**	17
**HPV_36**	3
**HPV_37**	4
**HPV_38**	4
**HPV_39**	23
**HPV_40**	8
**HPV_41**	-
**HPV_42**	22
**HPV_43**	11
**HPV_44 [formerly HPV 55]**	15
**HPV_45**	13
**HPV_47**	4
**HPV_48**	-
**HPV_49**	4
**HPV_50**	5
**HPV_51**	29
**HPV_52**	37
**HPV_53**	19
**HPV_54**	22
**HPV_56**	25
**HPV_57**	-
**HPV_58**	17
**HPV_59**	14
**HPV_60**	-
**HPV_61**	14

**HPV_62**	15
**HPV_63**	-
**HPV_65**	-
**HPV_66**	24
**HPV_67**	14
**HPV_68**	21
**HPV_69**	3
**HPV_70**	6
**HPV_71**	-
**HPV_72**	4
**HPV_73**	16
**HPV_74**	11
**HPV_75**	2
**HPV_76**	4
**HPV_77**	-
**HPV_78**	2
**HPV_80**	3
**HPV_81**	6
**HPV_82 [includes IS39]**	10
**HPV_83**	14
**HPV_84**	8
**HPV_85**	-
**HPV_86**	3
**HPV_87**	12
**HPV_88**	-
**HPV_89**	13
**HPV_90**	23
**HPV_91**	9
**HPV_92**	1
**HPV_93**	2
**HPV_94**	2
**HPV_95**	-
**HPV_96**	3
**HPV_97**	-
**HPV_98**	6
**HPV_99**	1
**HPV_100**	3
**HPV_101**	3
**HPV_102**	1
**HPV_103**	2
**HPV_104**	5
**HPV_105**	1
**HPV_106**	-
**HPV_107**	5
**HPV_108**	4
**HPV_109**	-

**HPV_110**	1
**HPV_111**	5
**HPV_112**	-
**HPV_113**	4
**HPV_114**	4
**HPV_115**	5
**HPV_116**	1
**HPV_117**	2
**HPV_118**	-
**HPV_119**	2
**HPV_120**	6
**HPV_121**	-
**HPV_122**	2
**HPV_123**	-
**HPV_124**	4
**HPV_125**	-
**HPV_126**	1
**HPV_127**	-
**HPV_128**	1
**HPV_129**	3
**HPV_130**	2
**HPV_131**	-
**HPV_132**	-
**HPV_133**	2
**HPV_134**	5
**HPV_135**	5
**HPV_136**	4
**HPV_137**	1
**HPV_138**	-
**HPV_139**	-
**HPV_140**	-
**HPV_141**	-
**HPV_142**	3
**HPV_143**	-
**HPV_144**	2
**HPV_145**	1
**HPV_146**	-
**HPV_147**	6
**HPV_148**	2
**HPV_149**	1
**HPV_150**	1
**HPV_151**	2
**HPV_152**	-
**HPV_153**	-
**HPV_154**	1
**HPV_155**	-

**HPV_156**	1
HPV_157	1
HPV_158	-
**HPV_159**	2
**HPV_160**	-
**HPV_161**	2
**HPV_162**	2
**HPV_163**	4
**HPV_164**	-
**HPV_165**	2
**HPV_166**	2
**HPV_167**	-
**HPV_168**	1
**HPV_169**	-
**HPV_170**	1
**HPV_171**	-
**HPV_172**	1
**HPV_173**	-
**HPV_174**	-
**HPV_175**	2
**HPV_178**	-
**HPV_179**	5
**HPV_180**	1
**HPV_184**	1
**HPV_197**	1
HPV_199	2
HPV_200	1
HPV_201	2
HPV_202	-
HPV_204	-
HPV_205	-
HPV_209	1
**HPV_mCG2**	-
**HPV_mCG3**	-
**HPV_HPV_mCH2**	1
HPV_mDysk1	1
HPV_mDysk2	1
HPV_mDysk3	1
HPV_mDysk5	-
HPV_mDysk6	-
HPV__mEV03c05	-
HPV__mEV03c09	-
HPV__mEV03c104	3
HPV__mEV03c188	1
HPV__mEV03c212	-
HPV__mEV03c40	2

HPV__mEV03c434	1
HPV__mEV03c45	1
HPV__mEV03c60	2
HPV__mEV06c107	-
HPV__mEV06c118	-
HPV__mEV06c12b	-
HPV__mEV07c367	-
HPV__mEV07c382	1
HPV__mEV07c390	1
**HPV_mFD1**	1
**HPV_mFD2**	1
**HPV_mFS1**	-
**HPV_mFi864**	1
HPV__mHIVGc36	2
HPV__mHIVGc70	-
HPV_mICB1	-
**HPV_mKC5**	-
**HPV_mKN1**	3
**HPV_mKN2**	2
**HPV_mKN3**	3
**HPV_mL55**	2
HPV__mLCOSOc196	-
HPV_mMTS1	-
HPV_mMTS2	-
HPV_mRTRX7	-
**HPV_mSD2**	7
**HPV_SE355**	1
HPV_mSE379	-
HPV_mSE383	-
HPV__mTVMBSFc09	1
HPV__mTVMBSGc529	1
HPV__mTVMBSGc2024	-
HPV__mTVMBSGc2450	-
HPV__mTVMBSHc13	-
HPV__mTVMBSHc33	-
HPV__mTVMBSWc141	-
HPV_mZJ01	-
HPV__md01c06	1
HPV__mdo1c02	1
HPV__mdo1c232	1
HPV__mga2c01	-
HPV__mga2c70	-
HPV__mm090c09	1
HPV__mm090c10	-
HPV__mm090c145	-
HPV__mm090c66	-

HPV__mm292c100	1
HPV__mm292c10	-
HPV__mm292c14	-
HPV__mm292c88	-
HPV__mw02c24a	4
HPV__mw03c65	3
HPV__mw07c34d	-
HPV__mw07c68b	-
HPV__mw07c74b	-
HPV__mw11C24	1
HPV__mw11C39	3
HPV__mw11C51	-
HPV__mw11c13	-
HPV__mw15c111	5
HPV__mw18c07	-
HPV__mw18c11d	-
HPV__mw18c134	2
HPV__mw18c25	-
HPV__mw18c39	-
HPV__mw20c01a	-
HPV__mw20c01b	-
HPV__mw20c02c	-
HPV__mw20c03a	-
HPV__mw20c04	1
HPV__mw20c08a	2
HPV__mw20c09	-
HPV__mw20c10a	-
HPV__mw21c693	-
HPV__mw22c09	-
HPV__mw23c08c	-
HPV__mw23c101c	-
HPV__mw23c77	3
HPV__mw27c04c	2
HPV__mw27c157c	2
HPV__mw27c39c	3
HPV__mw27c52c	-
HPV__mw34c04a	-
HPV__mw34c11a	1
HPV__mw34c14a	-
HPV__mw34c28a	-
HPV__mw34c34a	1
HPV__mwg1c05	2
HPV__mwg1c09	-
